# Gait pattern after electromechanically-assisted gait training with the Hybrid Assistive Limb and conventional gait training in sub-acute stroke rehabilitation—A subsample from a randomized controlled trial

**DOI:** 10.3389/fneur.2023.1244287

**Published:** 2023-10-11

**Authors:** Anneli Wall, Susanne Palmcrantz, Jörgen Borg, Elena M. Gutierrez-Farewik

**Affiliations:** ^1^Department of Rehabilitation Medicine Stockholm, Danderyd Hospital, Stockholm, Sweden; ^2^Department of Clinical Sciences, Danderyd Hospital, Karolinska Institutet, Stockholm, Sweden; ^3^KTH MoveAbility Lab, Department of Engineering Mechanics, Royal Institute of Technology, Stockholm, Sweden; ^4^Department of Women's and Children's Health, Karolinska Institutet, Stockholm, Sweden

**Keywords:** neurorehabilitation, robotics, exoskeleton, intervention, gait retraining

## Abstract

**Introduction:**

Electromechanically-assisted gait training has been introduced in stroke rehabilitation as a means to enable gait training with a large number of reproducible and symmetrical task repetitions, i.e. steps. However, few studies have evaluated its impact on gait pattern functions. This study includes persons with no independent ambulation function at the start of a 4-week neurorehabilitation period in the sub-acute phase after stroke. The primary aim of the study was to evaluate whether the addition of electromechanically-assisted gait training to conventional training resulted in better gait pattern function than conventional training alone. The secondary aim was to identify correlations between overall gait quality and standardized clinical assessments.

**Participants and methods:**

Seventeen patients with no independent ambulation function who participated in a Prospective Randomized Open Blinded End-point study in the sub-acute phase after stroke were randomized into two groups; one group (*n* = 7) to undergo conventional training only (CONV group) and the other group (*n* = 10) to undergo conventional training with additional electromechanically-assisted gait training (HAL group). All patients were assessed with 3D gait analysis and clinical assessments after the 4-week intervention period. Overall gait quality as per the Gait Profile Score (GPS), as well as kinematic, and kinetic and other spatiotemporal metrics were collected and compared between intervention groups. Correlations between biomechanical and clinical outcomes were evaluated.

**Results:**

Both the CONV and HAL groups exhibited similar gait patterns with no significant differences between groups in any kinematic, kinetic parameters or other spatiotemporal metrics. The GPS for the paretic limb had a median (IQR) of 12.9° (7.8°) and 13.4° (4.3°) for the CONV and HAL groups, respectively (*p* = 0.887). Overall gait quality was correlated with independence in walking, walking speed, movement function and balance. We found no added benefit in gait pattern function from the electromechanically-assisted gait training compared to the conventional training alone.

**Discussion:**

This finding raises new questions about how to best design effective and optimal post-stroke rehabilitation programs in patients with moderate to severe gait impairments to achieve both independent walking and optimal gait pattern function, and about which patients should be in focus in further studies on the efficacy of electromechanically-assisted gait training.

**Clinical trial registration:**

The study was retrospectively registered at ClinicalTrials.gov, identifier (NCT02410915) on April 2015.

## Introduction

Normal gait depends on sensory-motor neural networks at spinal and supraspinal levels (1, 2) and is often impaired by cerebral stroke, causing a hemiparetic gait pattern characterized by asymmetry and decreased walking speed (3, 4). Although walking speed often improves spontaneously over time and with rehabilitation interventions (3), asymmetric and compensatory gait patterns tend to remain (5). These gait patterns might incur an increased risk of musculoskeletal complications and falls, and increased energy expenditure in the long term (3–5). Gait interventions aiming at improving gait symmetry after stroke have recently been suggested (6–8) though predominantly in a chronic stroke population.

Current evidence accentuates the importance of intensive, repetitive, and task-specific training of movement functions to drive neuroplasticity (9) and support motor relearning (10–12) after stroke. In addition, to improve gait function, the dose (number of steps), intensity (heart rate and/or walking speed) and variability of task training are considered important (11, 13).

Electromechanically-assisted gait training has been introduced in rehabilitation to facilitate higher dosage (i.e. more steps) per training session, more reproducible and symmetrical gait than through manual support from a therapist, and potentially earlier start of intensive gait training after stroke despite even severe impairment (14). A recent review reported beneficial effects on independence in walking after electromechanically-assisted gait training combined with physiotherapy (14). However, data describing the effects on gait patterns after such training are limited and when presented, often describe only a few spatiotemporal parameters, specifically walking velocity and cadence (14). More knowledge in this area can elucidate to what extent improvements in gait pattern are related to walking performance.

Several types of robotic exoskeletons are currently in clinical use or under development. Most of these support gait training by use of automatic motions, such as the Lokomat (Hocoma, Volketswil, Switzerland) (15). However, timely assistance and active patient participation during training have been proposed as important for promoting adequate motor learning and activity-dependent neuroplasticity (16, 17). As such, rehabilitation robotic devices involving a user-driven control approach may be more beneficial than those with an autonomous approach.

**Figure 1:**
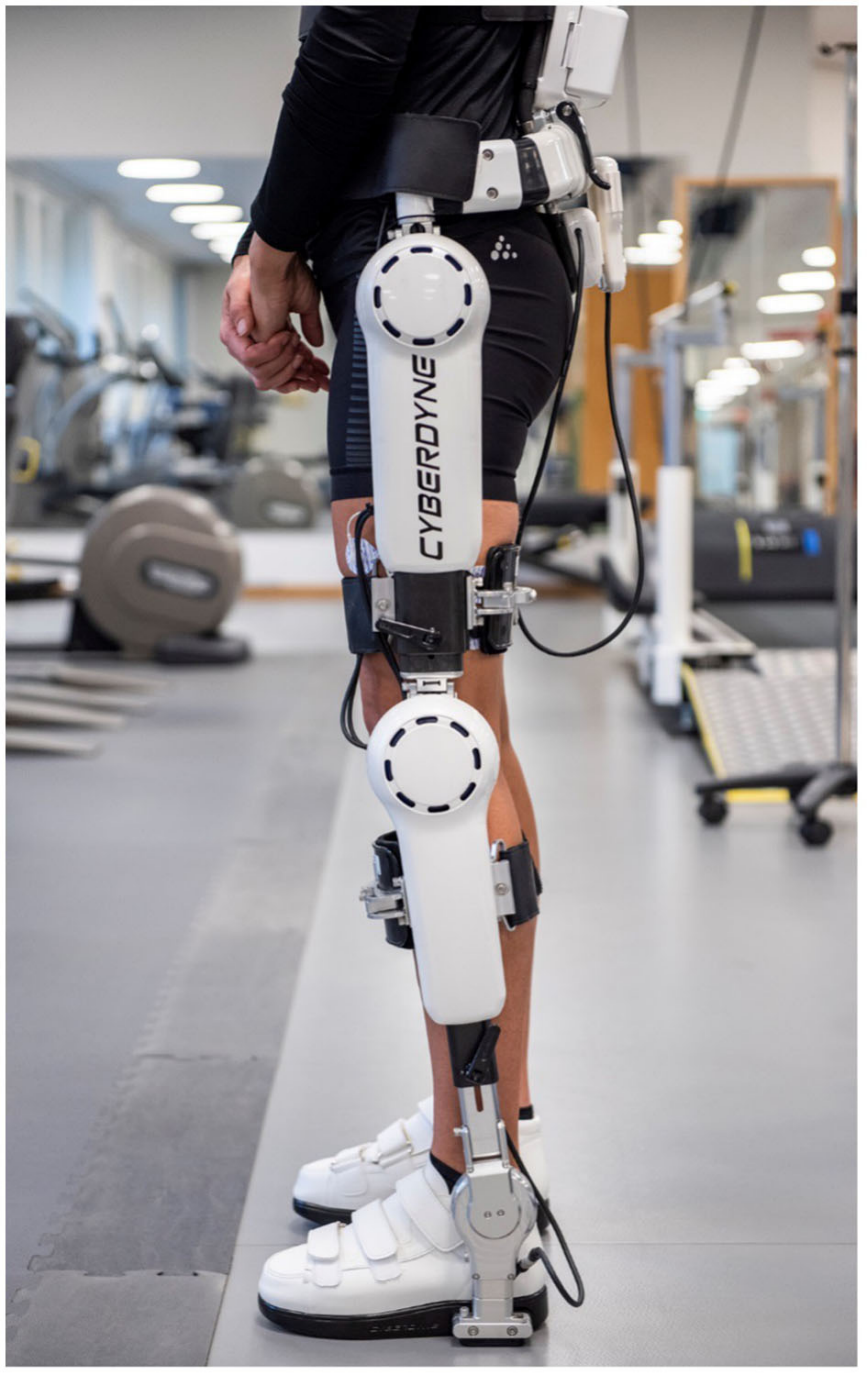
The Hybrid Assistive Limb (HAL). Photographer: Johan Adelgren.

The Hybrid Assistive Limb (HAL, Figure 1) is an intention-based exoskeleton for supporting gait training, that allows both voluntary and autonomous control to support gait training (18, 19). Involuntary mode, motions are triggered by the user's muscle activation, as recorded by surface electrodes placed over the extensor and flexor muscles of the hip and knee. This technology enables even minimal muscle activity to initiate and assist voluntary movements (19). Earlier studies of HAL in clinical applications have indicated beneficial effects on independence in walking and walking speed (20). In a recent study including but not limited to the patients in the current study, we have shown that gait training with HAL in patients in the subacute stage after stroke enabled longer walking distances during HAL training sessions, but with no additional effect on independence in walking and walking speed after a 4-week intervention, compared to after conventional gait training in the subacute stage after stroke (21). However, potential effects on movement-related function such as gait pattern remain unknown. It has been reported that in patients in the chronic stage after stroke, 8 sessions of HAL training resulted in increased stride length and single limb support time on the affected side (21). Yet, whether HAL training affects kinematics and kinetics of gait, as compared to after conventional gait training is still unknown.

Thus, the primary aim of the study was to evaluate whether the addition of electromechanically-assisted gait training to conventional training resulted in better gait pattern function than conventional training alone. A secondary aim was to evaluate whether correlations between overall gait quality and standardized clinical assessments exist, in order to better identify which clinical presentation variables may be related to, or serve as a proxy for, a greater potential for gait pattern function improvement in this patient group.

## Methods

### Design

This study is part of a Prospective Randomized Open Blinded End-Point Study, approved by the Stockholm Ethical Review Board (November 13, 2013, ref. 2013/1807-31/2 and October 6, 2014, ref. 2014/1633-32), referred to as “the main study”. The main study protocol can be found at ClinicalTrials.gov (Identifier: NCT02410915) and has been described in detail in previous publication (21). Among patients in the main study, this study includes the 17 patients who were assessed with 3D gait analysis.

### Intervention

All patients participated in conventional team-based training provided by a multidisciplinary team, following current evidence and best practice for inpatient rehabilitation after stroke (performed daily, 5 days/week). Directly after inclusion, block-randomization was performed by a nurse who was otherwise not involved in the study into (1) conventional training or (2) HAL-training in addition to conventional training.

The conventional training included physiotherapy training that could be comprised of training of movement functions in both the upper and lower extremity, balance, and gait. The gait training could include stepping, weight shifting, over ground walking as well as the use of treadmill with or without body-weight support.

Individualized HAL-training was planned for 4 days/week for 4 weeks (i.e. 16 sessions) with the single-leg version of HAL, in voluntary mode, on a treadmill with body-weight support. To obtain a symmetrical gait pattern as close to normal gait as possible, the therapist continuously optimized HAL-settings based on observational gait analysis during each session.

### Participants

**Figure 2:**
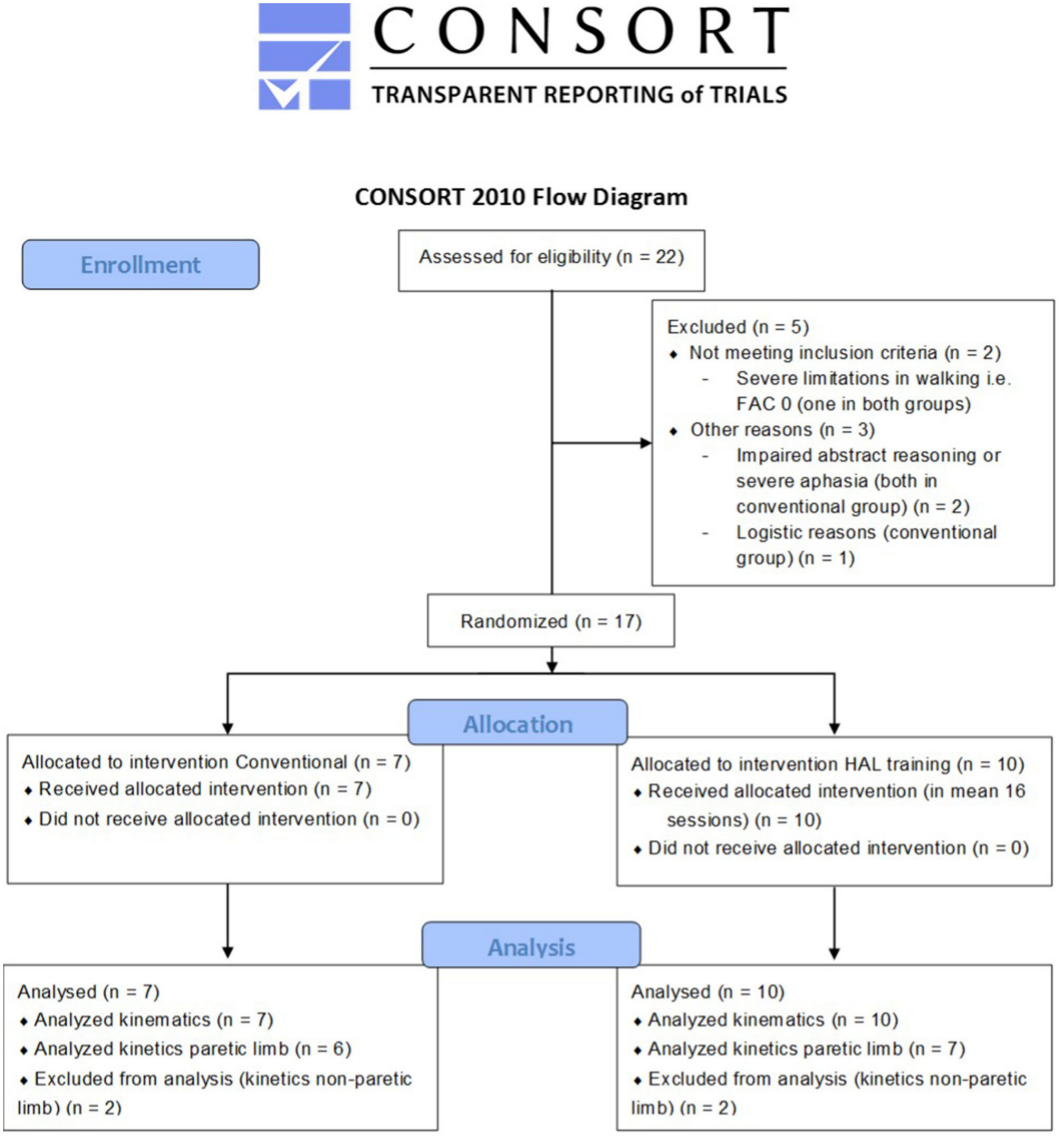
CONSORT 2010 flow diagram of this sub-study. FAC, Functional Ambulation Categories; HAL, Hybrid Assistive Limb. Kinematics data is available in all patients, but some kinetics data is missing; it was not possible to collect ground reaction force data on the paretic side in some patients due to interference from walking aids.

Patients in the main study were recruited from a sub-acute inpatient rehabilitation unit (Danderyd Hospital, Stockholm, Sweden) admitting patients aged 18–67 years, during February 2014 and May 2017. Inclusion criteria were:< 8 weeks since onset of ischemic or hemorrhagic stroke (verified by computerized tomography and/or magnetic resonance imaging), Functional Ambulation Categories (FAC) score of 0–1 and thus need for continuous manual support to walk due to lower extremity paresis, ability to maintain a sitting posture for >5 min, sufficient postural control to allow an upright position in standing with aids and/or manual support, ability to understand training instructions, ability to understand written and oral study information to express informed consent, and a body size compatible with the HAL-suit. Exclusion criteria included cerebellar stroke, primary subarachnoid bleeding, lower limb contracture that restricted gait movements, cardiovascular or other somatic condition incompatible with intensive gait training, and severe, contagious infections. The main study included 36 patients, of whom 32 completed the intervention. After inclusion of the 11th patient, gait analysis (GA) was added as an outcome evalution method, at which point consecutive sampling for the current study began. Of the 22 patients recruited after GA was included, 17 patients were able to walk barefoot over a 10-meter walkway with or without a walking aids after the 4-week intervention period, and were thus included in the current study (Figure 2). They comprise the conventional training group (CONV, *n* = 7) and HAL-training in addition to the conventional training group (HAL, *n* = 10). It was not possible to perform GA at the start of the intervention due to the patients' severe walking limitations at inclusion.

At inclusion in the current study, patients exhibited moderate-to-severe stroke; 57% (4/7) in the CONV group and 60% (6/10) in the HAL group had FAC 0 (non-functional ambulation), all others FAC 1 (dependence on continuous physical assistance for support and ambulation). There were no significant differences in patient characteristics between the CONV and the HAL groups (Table 1). The GA was performed at a mean of 66 (SD 15) days after stroke onset. All patients except one (in the CONV group) used a walking aid, most commonly a hemi-walker, during GA.

**Table 1:** Characteristics of patients with collected kinematics and kinetics.

	**Kinematics only**		**Kinematics** + **kinetics**	
	**HAL**	**CONV**	**HAL**	**CONV**
*N*	10	7	7	6
Men/women	8/2	6/1	6/1	6/0
Age, years: mean (SD)	52 (11)	50 (13)	50 (13)	49 (13)
Diagnosis: hemorrhage/infarction	3/7	5/2	3/4	4/2
Paretic side: left/right	8/2	5/2	6/1	4/2
Days from stroke to inclusion: mean (SD)	32 (14)	42 (16)	34 (16)	42 (18)
NIHSS at inclusion in main study: median (IQR)	11 (8.75, 13)	12 (11, 13)	9 (8, 13)	12 (10.25, 14.25)

CONV, Conventional group; HAL, HAL group; NIHSS, NIH, Stroke Scale (describes the stroke severity).

### Data collection

The GA was performed after the intervention period with a motion capture system (Vicon MX40, Oxford, UK) and two force plates (Kistler, Winterthur, Switzerland). Twenty-seven reflective markers were placed over anatomical landmarks on the pelvis, legs, trunk and head according to a conventional model (Vicon Plug-in-Gait) (22). Patients walked barefoot with their walking aid if needed, at a self-selected speed over a 10-meter walkway with supervision or, if needed, minimal manual support from a physiotherapist. The marker placement was performed by one examiner, with assistance from one of two other experienced examiners, all using the same protocol but not blinded to the intervention allocation.

The blinded standardized clinical assessments analyzed in the current study were conducted before and after the intervention period, and included the FAC, which evaluates independence in walking on a six-grade ordinal scale ranging from non-functional walking (FAC 0) to independent walking on non-level surfaces (FAC 5) (23), the 2 Minute Walk Test (2MWT) (24), the Berg Balance Scale (BBS) (25), and the Fugl-Meyer Assessment lower extremity motor (FMA-LE Motor) and sensory (FMA-LE Sensory) function scales (26). The National Institutes of Health Stroke Scale (NIHSS) for stroke severity (27) was also used at inclusion in the main study to assess stroke severity.

The primary outcome in this study was overall gait quality assessed as the Gait Profile Score (GPS) for the paretic and non-paretic leg separately (28). Secondary outcomes included correlations between GPS and clinical assessments, as well as kinematics and positive joint work during walking.

### Data processing

Kinematic data were calculated from three gait cycles per side for each patient (only two cycles per side were available for two patients). The gait cycles used were considered sufficient and representative after visual inspection of video and curve correspondence, in accordance with the standard practices of GA. Kinetics in the paretic limb were successfully collected in 13 patients with a mean of two cycles per patient. Kinetics in the non-paretic limb were only successfully collected in four patients (Figure 2) and are therefore not discussed here.

Quantitative parameters of kinematics and kinetics, as well as GPS, were obtained for each gait cycle, and average parameters for each side (paretic and non-paretic) were computed. Other spatiotemporal metrics describing speed, step frequency and distance were non-dimensionalized according to Hof (29). Kinetics were normalized to body mass, and positive joint work was calculated as the positive integral of joint power. Reference values were obtained from a group of 81 non-disabled subjects (Controls), with 46% men and a mean age of 45 (SD 18) years.

The GPS quantifies the magnitude of gait kinematics deviation in degrees in the lower body (GPS-LB) or whole body (lower body and trunk, GPS-WB) wherein larger GPS indicate greater deviation from a normative dataset. More specifically, the GPS-LB is the average Euclidean difference in degrees from the normative dataset of the pelvis and hip angles in all three planes, the sagittal knee and ankle angles and the transverse foot progression angle. The GPS-WB further includes the trunk angle deviation in all three planes. The GPS can be divided into Gait Variable Scores (GVSs) to show each gait variable's deviation throughout the gait cycle (28). The Movement Analysis Profile illustrates to what extent each of the parameters contribute to the GPS score (29). The GPS has been extensively used and found reliable in several patient groups (30) and was recently found reliable and suggested as useful for assessing gait quality post-stroke (31).

### Statistical analysis

Descriptive statistics and tests for normality were performed using Shapiro-Wilk's test, skewness, boxplots and QQ-plots. Calculations were performed in SPSS (IBM SPSS Statistics 22) with 2-tailed significant levels of *p* < 0.05 or, in case of multiple testing, *p* < 0.01.

Group differences were tested with Mann-Whitney U-test for age, stroke severity, GPS, GVSs, and non-normally distributed kinematics, kinetics, and other spatiotemporal variables as well as for data on training intensity and distances and on FAC, BBS, FMA-LE, 2MWT change scores; with independent sample *t*-test for time to inclusion and normally-distributed kinematics, kinetics and other spatiotemporal variables; and with the Fisher exact test for gender, diagnosis, and paretic side. Differences within groups (paretic vs. non-paretic side) for GPS and GVSs, and FAC, BBS, FMA-LE, 2MWT (baseline vs. post-intervention) were tested with Wilcoxon signed-rank tests.

Correlations between clinical assessments (FAC, 2WMT, BBS, and FMA-LE Motor and Sensory) and overall gait quality, defined as GPS-LB on the paretic side (GPS-LBP), were computed for the whole (merged) group by Spearman's rank correlation (R_S_). Correlations were interpreted as little or no relationship for R_S_ ≤ 0.25, fair for R_S_ between 0.25 and 0.50, moderate to good for R_S_ between 0.50 and 0.75, and very good to excellent relationship for R_S_ ≥ 0.75 (32).

## Results

### HAL and conventional gait training

In the HAL group, seven patients performed all 16 HAL-sessions, two performed 15 sessions and one performed 14 sessions. The body-weight support provided was set to 30% of the patient's body weight at the first session, and had at the last session been decreased to an average of 19% (SD 5%) body weight. During HAL training, patients walked a median (Interquartile range IQR) distance of 519 (329, 741) meters per session, with 219 (114, 316) and 760 (548, 852) meters during the first and last session, respectively.

Data on the conventional gait training were retrieved from the patients' medical records. Estimated distance walked during the conventional gait training sessions were recorded in 82% (HAL group 90%, CONV group 70%) of the records (missing fully for one patient in the CONV group). There was a significant difference in the total number of gait training sessions (both conventional and HAL gait training) performed in each group, wherein more sessions were performed in the HAL group [median (IQR): HAL group 22.5 (19, 24.5), CONV group 12.5 (9, 21.5), *p* = 0.022], and a significant difference in the median number of conventional gait training sessions performed in the two groups, wherein more sessions were performed in the CONV group [HAL group 7.0 (4.5, 9.25), CONV group 12.5 (9, 21.5), *p* = 0.042]. There was a significant difference between groups in the median distance walked during these conventional gait training sessions, wherein a greater distance was walked in the CONV group [HAL group 24.5 (15, 47.5), CONV group 95.0 (47.5, 210), *p* = 0.018].

### Overall gait quality

**Figure 3:**
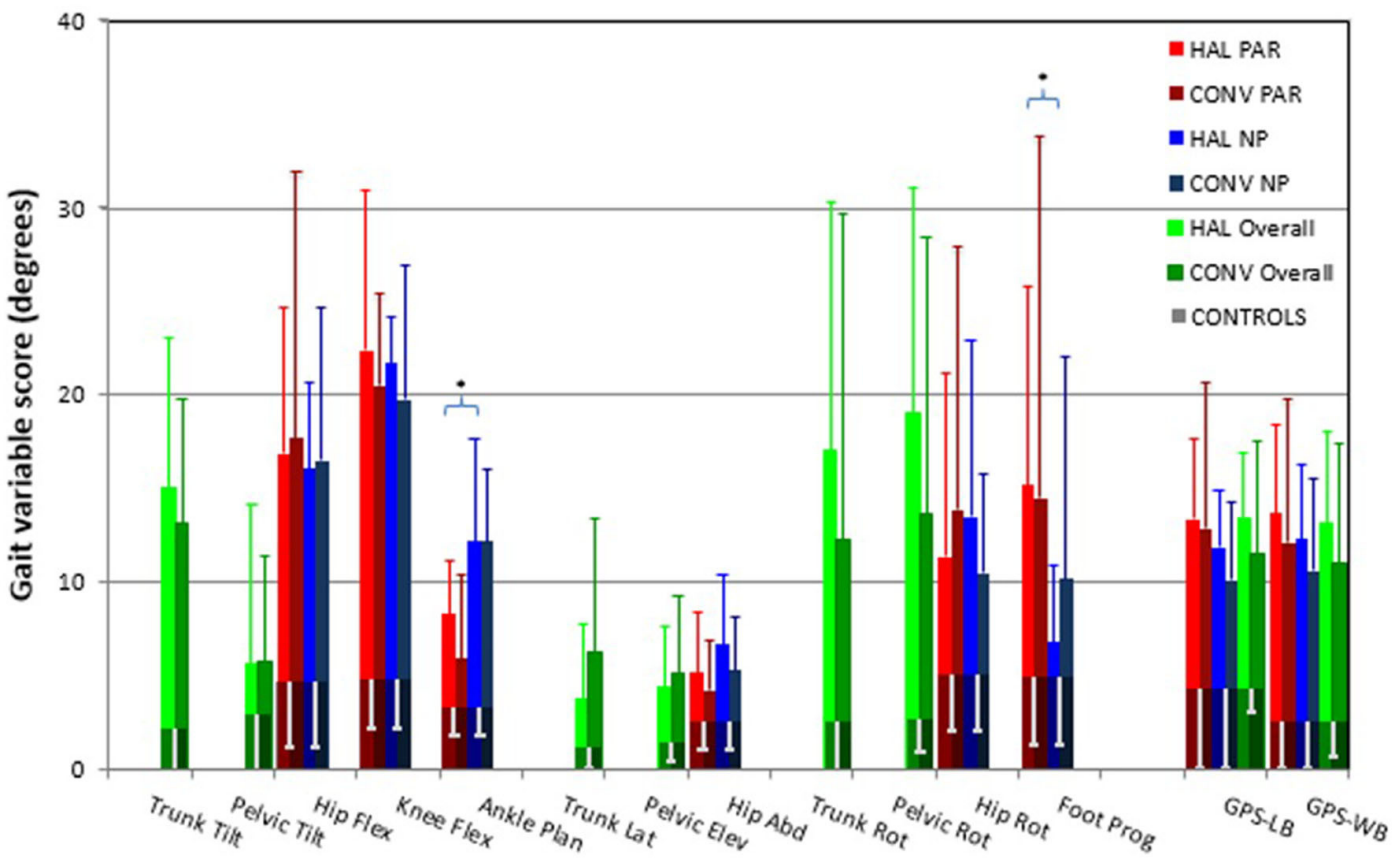
The Movement Analysis Profile with GPS (gait profile score) and GVSs (gait variable scores) presented as medians and interquartile ranges for both groups. PAR, paretic limb; NP, non-paretic limb; HAL, HAL group; CONV, Conventional group; CONTROLS, non-disabled subject group; GPS-LB, GPS lower body; GPS-WB, GPS whole body (lower body and trunk); Hip Flex, hip flexion/extension; Knee Flex, knee flexion/extension; Ankle Plan, ankle dorsiflexion/plantarflexion; Trunk Lat, trunk lateral flexion; Pelvic Elev, pelvic obliquity; Hip Adb, hip adduction/abduction; Trunk Rot, trunk rotation; Pelvic Rot, pelvic rotation; Hip Rot, hip rotation; Foot Prog, foot progression. *Indicates significant difference between limbs in the HAL group for ankle dorsi/plantarflexion (*p* = 0.005) and foot progression (*p* = 0.007).

There were no significant differences in GPS or any GVSs between the CONV group and the HAL group for either the paretic or the non-paretic leg (Figure 3). The median (IQR) GPS-LBP was 12.9 (7.8) in the CONV group and 13.4 (4.3) in the HAL group (*p* = 0.887).

### Kinematic, kinetic, and other spatiotemporal parameters

**Figure 4:**
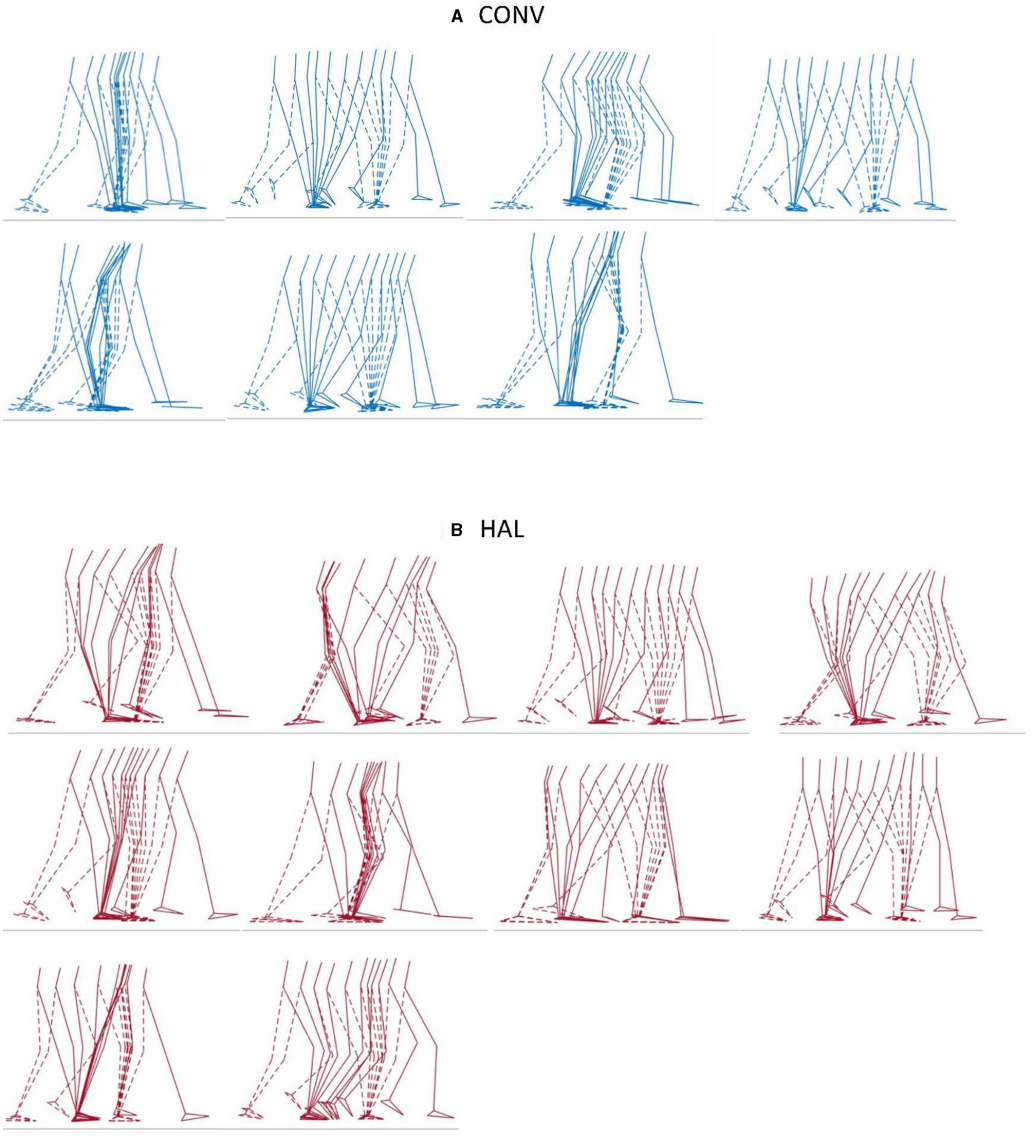
Illustrations of one gait cycle for each patient in the **(A)** CONV (*n* = 7, blue) group and **(B)** HAL (*n* = 10, red) group. The paretic leg is displayed with solid lines, and the non-paretic leg with dotted lines.

All patients demonstrated impaired gait kinematics and step spatiotemporal asymmetry, with no significant differences between groups after the intervention period (Table 2). Gait patterns within and between groups were heterogenous (Figure 4). Kinematics from a gait cycle from the paretic side for each patient in each intervention group is illustrated in Supplementary Figure 1, and kinetics, in Supplementary Figure 2.

**Table 2:** Spatiotemporal metrics, kinematic, and kinetic data of the paretic (P) and non-paretic (NP) sides.

**Parameter**	**HAL**	**CONV**	**Controls**	***p***
**Spatiotemporal metrics**	***n** =* **10 mean (99% CI)**	***n** =* **7 mean (99% CI)**	***n** =* **81 mean (99% CI)**	**HAL vs.CONV**
Cadence P Cadence NP (Steps/min)	41.7 (20.5, 63.0) 43.6 (22.4, 64.8)	50.3 (18.8, 81.8) 50.0 (16.8, 83.3)	119.6 (116.9, 122.2)	0.431 0.563
NN walking speed: mean of P and NP	0.07 (0.02, 0.12)	0.09 (−0.02, 0.21)	0.47 (0.45, 0.48)	0.740
NN stride length: mean of P and NP	0.58 (0.38, 0.79)	0.64 (0.19, 1.09)	1.54 (1.51, 1.57)	0.678
NN step length P NN step length NP	0.36 (0.23, 0.49) 0.22 (0.01, 0.35)	0.41 (0.26, 0.56) 0.22 (−0.11, 0.55)	0.76 (0.75, 0.78)	0.436 0.977
Stride time: mean of P and NP (s)	3.54 (1.71, 5.37)	2.99 (0.65, 5.32)	1.01 (0.99, 1.03)	0.417
Swing time P Swing time NP (% Gait cycle)	26.5 (14.8, 38.1) 13.1 (5.6, 20.6)	28.5 (13.9, 43.2) 14.5 (−1.1, 30.0)	40.4 (40.0, 40.8)	0.711 0.813
Stance time P Stance time NP (% Gait cycle)	73.5 (61.9, 85.2) 86.9 (79.4, 94.4)	71.6 (57.2, 86.0) 85.5 (70.0, 101.1)	59.6 (59.2, 60.0)	0.728 0.813
Single support P Single support NP (% Gait cycle)	12.9 (4.9, 20.9) 27.2 (15.5, 39.0)	14.7 (−0.1, 29.5) 28.6 (14.4, 42.8)	40.0 (39.6, 40.4)	0.962 0.802
Double support: mean of P and NP (% Gait cycle)	60.3 (42.9, 77.7)	57.1 (29.3, 84.8)	19.6 (18.9, 20.3)	0.637
**Kinematics**	***n** =* **10**	***n*** =**7**		
Hip flexion range P Hip flexion range NP (°)	22.4 (12.0, 32.9) 33.9 (25.0, 42.9)	22.5 (3.3, 41.7) 35.5 (26.0, 45.1)	45.5 (44.3, 46.6)	0.997 0.692
Hip flexion peak in swing P Hip flexion max in swing NP (°)	31.0 (19.0, 42.9) 35.7 (27.3, 44.0)	32.9 (25.8, 40.1) 34.0 (29.2, 38.9)	30.4 (28.8, 32.1)	0.681 0.581
Knee flex range P Knee flex range NP (°)	31.2 (20.1, 42.3) 41.7 (32.6, 50.8)	29.6 (15.0, 44.2) 44.1 (26.9, 61.2)	58.2 (56.9, 59.4)	0.763 0.646
Knee flex peak in swing P Knee flex peak in swing NP (°)	27.5 (13.7, 41.3) 49.3 (38.9, 59.7)	27.1 (11.0, 43.2) 48.3 (34.0, 62.6)	55.7 (54.5, 56.9)	0.949 0.843
Ankle sagittal range P Ankle sagittal range NP (°)	19.0 (13.4, 24.7) 23.1 (16.6, 29.6)	18.6 (6.5, 30.6) 23.3 (14.5, 32.1)	27.4 (26.2, 28.7)	0.891 0.943
Ankle sagittal at IC^*^ P Ankle sagittal at IC^*^ NP (°)	−5.3 (−13.1, 2.4) 3.8 (−2.6, 10.2)	−8.7 (−14.6, −2.9) 5.4 (−4.1, 14.9)	−0.5 (−1.4, 0.4)	0.297 0.618
**Kinetics (Paretic leg)**	***n*** =**7**	***n*** =**6**		
Hip positive work (J/kg)	0.08 (0.05, 0.12)	0.11 (−0.05, 0.26)	0.20 (0.17, 0.23)	0.945
Knee positive work (J/kg)	0.05 (0.02, 0.09)	0.06 (0.00, 0.13)	0.11 (0.09, 0.12)	0.484
Ankle positive work (J/kg)	0.09 (−0.01, 0.19)	0.09 (−0.06, 0.25)	0.25 (0.22, 0.28)	0.945

Data presented as means and 99% confidence intervals and shown for the (randomly-chosen) left side of the controls as a reference.

HAL, HAL group; CONV, Conventional group; Controls, non-disabled subject group; P, paretic leg; NP, non-paretic leg; NN, non-dimensionalized; IC, initial contact; flex, flexion.

* Negative (-) degrees indicates plantarflexion.

**Figure 5:**
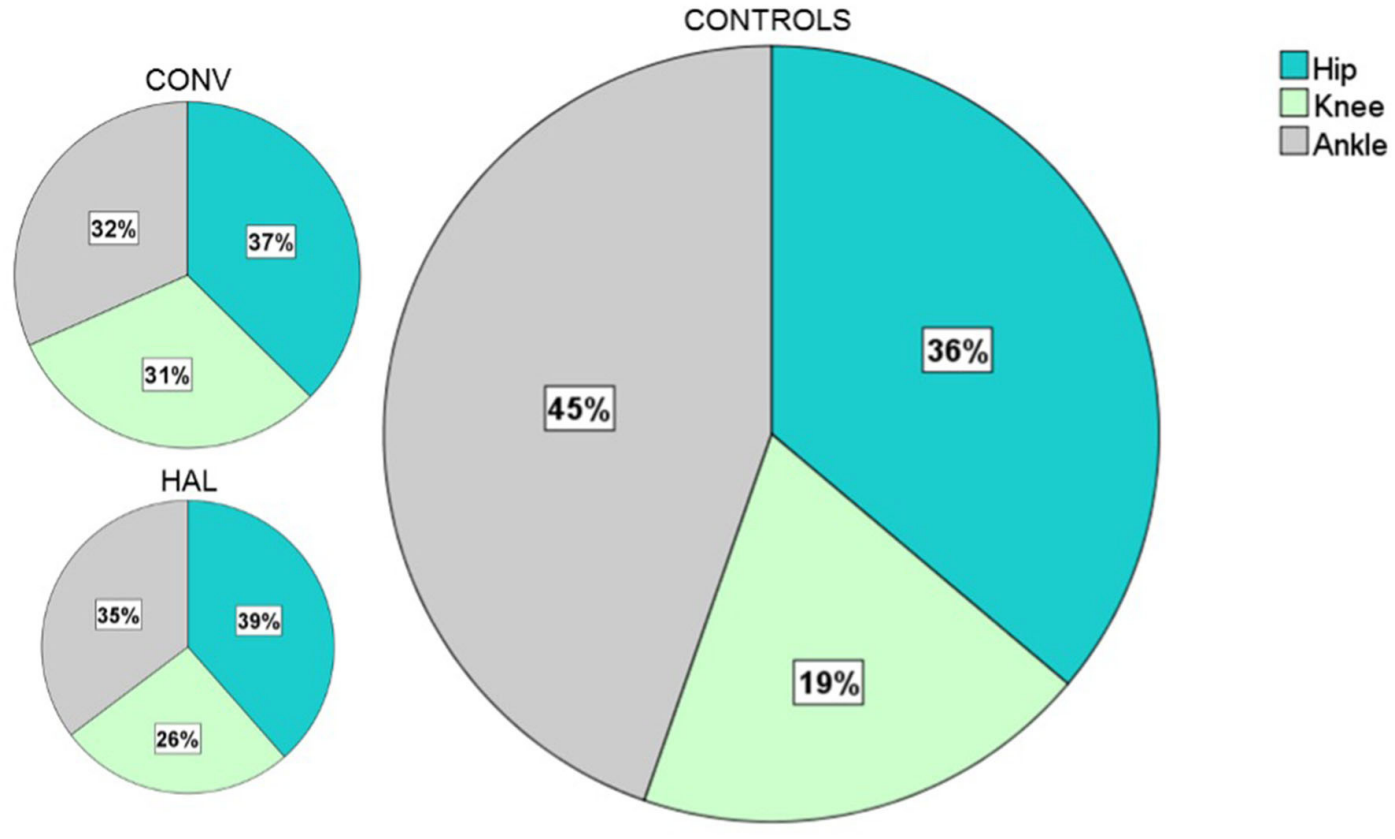
Mean contribution of positive work for Hip, Knee and Ankle on the paretic side. The figures are scaled to a percent of overall positive lower limb joint work in which controls represent 100%. The Conventional and HAL groups had an overall positive work of 46% and 39% that of the controls, respectively. The overall positive joint work for the HAL group was 85% that of the Conventional group (non-significant). The proportional contributions of each joint to the total positive work were similar in both groups (Hip p = 0.870, Knee p = 0.594, Ankle p = 0.708). HAL, HAL group; CONV, Conventional group; CONTROLS, non-disabled subject group.

The proportional contributions of each joint to total positive work were similar in both groups (no significant difference) and much lower than in controls (Figure 5).

### Clinical assessments and overall gait quality

Between the baseline and post-intervention assessments, FAC, 2MWT and BBS improved significantly in both groups, and FMA-LE Motor improved in the HAL group. However, there were no significant differences in change scores (difference from baseline to post-intervention) in any of these clinical outcomes between CONV and HAL groups (Table 3). After intervention, 43% (3/7) in the CONV group and 50% (5/10) in the HAL group were considered physically independent walkers (FAC 3–5). All except one patient (HAL group) had impaired sensory function (FMA-LE Sensory < 12).

**Table 3:** Clinical outcomes at baseline and post-intervention.

**Clinical assessments [Median (IQR)]**	**HAL (*****n** =* **10)**			**CONV (*****n** =* **7)**			**Changes HAL vs. CONV**
	**Pre**	**Post**	**Δ**	**Pre**	**Post**	**Δ**	p^**^
FAC	0 (0, 1)	2.5 (1.75, 3)	2^*^ (1, 2.25)	0 (0, 1)	2 (2, 3)	2^*^ (2, 3)	0.536
2MWT (m)	4.75 (0, 7)	20.8 (7.25, 34.25)	14.8^*^ (7.25, 28)	4 (0, 14)	22.5 (15, 67)	19.5^*^ (8.5, 63)	0.417
BBS	9 (5.5, 13.5)	28.5 (11.75, 43)	10^*^ (6.75, 34)	14 (7, 22)	26 (13, 39)	12^*^ (4, 23)	0.475
FMA-LE motor	8 (5.5, 15)	16.5 (4.75, 21)	3^*^ (0.75, 9.75)	14 (7, 24)	16 (7, 27)	0 (−1, 3)	0.088
FMA-LE sensory	6 (2.25, 11)			7 (4, 10)			

* p < 0.05 according to Wilcoxon.

** p according to Mann-Whitney.

Values were not normally distributed and are therefore presented as median [interquartile range].

HAL, HAL group; CONV, Conventional group; Δ, change from baseline to post intervention; FAC, Functional ambulation categories; 2MWT, 2 Min Walk Test; FMA-LE, Fugl-Meyer Assessment lower extremity Motor (34 p) and Sensory domains (12 p).

**Figure 6:**
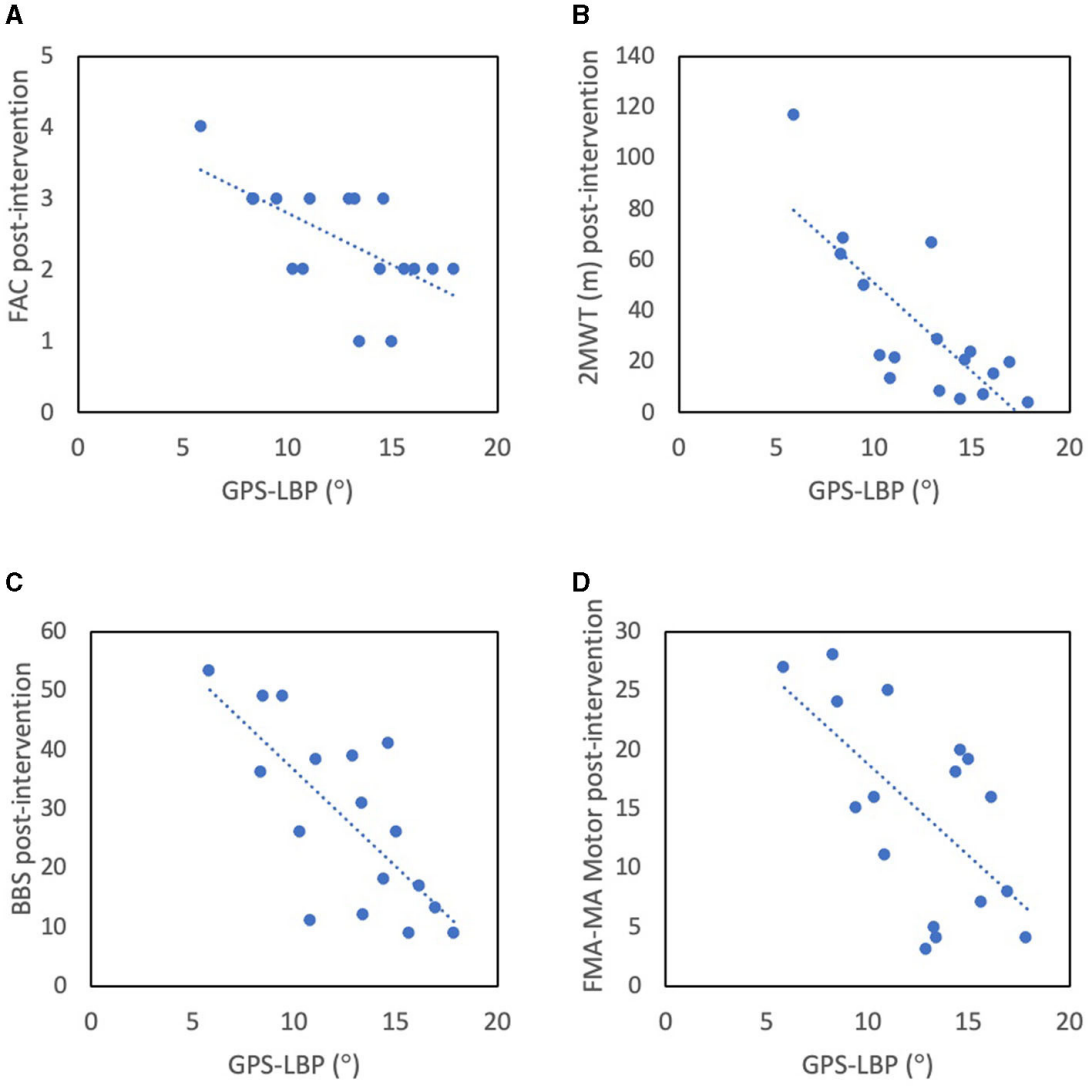
Correlations between gait quality, presented as GPS-LB in the paretic side, and clinical assessment outcomes post-intervention: **(A)** FAC, **(B)** 2MWT, **(C)** BBS, and **(D)** FMA-LE Motor, for the entire study group (*n* = 17). GPS-LBP, Gait Profile Score (°) in lower body in paretic side; FAC, Functional Ambulation Category indicating gait independence; BBS, Berg Balance Score; FMA-LE Motor, Fugl-Meyer Assessment Lower Extremity motor function scale.

Due to the small study sample and homogeneity in results between the two groups, the correlations between clinical assessment and overall gait quality were computed for one merged group (*n* = 17). We found a moderate-to-good and significant correlation between overall gait quality (GPS-LBP) and independence in walking (FAC) (R_S_ = −0.625, *p* = 0.007), walking speed/endurance (2MWT) (R_S_ = −0.733, *p* = 0.001), balance (BBS) (R_S_ = −0.685, *p* = 0.002) and movement function (FMA-LE Motor) (R_S_ = −0.504, *p* = 0.039, Figure 6), wherein higher GPS, i.e. larger deviation from the normative database and thus lower gait quality, correlates with lower clinical assessment variables. No correlation was found between GPS-LBP and sensory function (FMA-LE Sensory) (R_S_ = 0.055, *p* = 0.832).

## Discussion

This study is the first to compare gait pattern functions after conventional training alone or combined with electromechanically-assisted gait training with HAL in the sub-acute phase after stroke. The study was designed to specifically determine whether additional training with HAL had any added benefit to conventional gait training. It is also among relatively few studies that include gait quality outcome variables on this patient population, whose poor pre-intervention ambulatory function makes it impossible to evaluate gait before intervention.

Despite differences in training, we found no differences between the intervention groups in any kinematic or kinetic or other spatiotemporal parameters. The gait patterns in each intervention group were heterogenous (Figure 4 and Supplementary Figures 1, 2), and no clear patterns of compensatory mechanisms could be observed; it was for this reason that overall gait quality was used as an outcome measure. Among clinical outcomes, the FME-LE Motor score improved in the HAL group but not in the CONV group, though neither group's improvement achieved the reported minimum clinically important difference (MCID) of 10 (33). Furthermore, as the median baseline value was higher in the CONV group, and as the post-intervention value was similar in both groups, it is likely that this difference was more a consequence of randomization than of intervention group. The other clinical outcomes improved in both groups, wherein the BBS MCID, reported as 6 (34), and the FAC MCID, reported as 1 (23), were achieved in both groups. Improvements in 2MWT did not reach the MCID reported as 37.2 m (35), in either group. There were no differences in change scores between the groups in any of the scores. In the study population as a whole, we identified correlations between overall gait quality (GPS-LBP) and clinical assessments of body function and activity, with moderate-to-good correlation with independence in walking (FAC), walking speed/endurance (2MWT), balance (BBS) and movement function (FMA-LE Motor). The patients in this study were dependent on assistance in walking and thus represented the patient population who reportedly can be expected to benefit most from electromechanically-assisted gait training in combination with conventional physiotherapy (14). Despite this, our findings could not corroborate any expected benefit regarding gait pattern functions. It is worth noting that in the main study we found no additional benefits of electromechanically-assisted gait training on functioning (such as independence in walking and walking speed), compared to after conventional gait training alone (21).

After stroke, improvement in movement-related functioning, including walking ability, can reflect both spontaneous processes and responses to interventions (36, 37). While *compensation* involves the use of alternative strategies, such as changes in muscle activation, timing, and kinematic patterns to perform a movement, *recovery* implies relearning to perform a movement with the same kinematic patterns as before stroke (36, 38). Whether improvements in movement-related functioning are achieved due to compensation or to recovery have yet to be distinguished (36, 39), and movement analysis is likely to be useful in clarifying this relationship. Few studies, however, have compared kinematics and kinetics after electromechanically-assisted gait training vs. conventional gait training, and none using HAL in particular, making comparisons difficult. Our results suggest that HAL-training according to the design applied here might not have an impact on gait patterns despite within-group improvements in walking performance.

Our results do not corroborate findings of Puentes et al. (40, 41), who reported that, in sub-acute stroke and post-decompression surgery due to ossification of the posterior longitudinal ligament, HAL training improved gait coordination (intersegmental coordination/symmetry) toward that of non-disabled controls. They suggest that early onset of HAL training can induce plasticity and true recovery of gait patterns, impeding establishment of compensatory gait movements (40). Beneficial effects on gait patterns have also been found in the chronic stage after stroke (21). However, none of those studies contain a control group, and whether the reported results could be obtained by evidence-based, non-compensatory, conventional gait training was not examined. In addition, patients in these cited studies were less impaired in gait at baseline that those in the current study, making pre-intervention assessment possible, in contrast to patients in the current study, whose more severe impairment made it impossible to perform a GA before intervention. A possible explanation for our conflicting findings might be that patients with moderate to severe gait impairments like those included in our study are restricted to using a stereotypical gait pattern to walk, and struggle to walk at all, at the expense of gait quality.

According to the GPS, gait pattern was impaired bilaterally, with similar deviations in the paretic and non-paretic limbs (13° and 10–12°, respectively). These deviations are similar to findings from Devetak et al. (31), who reported GPS of 11° and 13.5° in paretic and non-paretic limbs, respectively, in patients post-stroke. However, all patients in that study were physically independent in walking compared to only 47% (8/17) in our study sample. This indicates that patients in our study deviated to the same extent from normal gait as patients who are less dependent in walking, which indicates that gait quality alone might not explain variance in independence in walking.

During HAL-training, knee flexion was assisted during swing to obtain foot clearance with minimal compensatory movements. However, knee flexion in swing on the paretic side was similar in both groups and less than that in the non-paretic side and in the non-disabled subjects (Controls). This corroborates earlier observations suggesting that recovering pre-stroke joint kinematics might not be required for improved independence in walking and that a stiff-knee pattern might even be useful to accommodate walking (42). One could speculate that in these patients, who have severe limitations in walking, the use of compensatory movements is essential to be able to walk at all, and a relatively high GPS can thus be expected.

Our results suggest furthermore that the repetitive, reproducible and symmetrical gait pattern strived for during HAL-training was not transferred to symmetrical over-ground walking after the intervention period. Whether gait patterns are different in treadmill-walking compared to over ground walking in patients in the acute post-stroke phase has not yet been ascertained, to the best of our knowledge. However, in non-disabled individuals and in chronic stroke patients, treadmill-walking is reported to be less variable (43, 44), which might affect the ability to transfer locomotor skills to over-ground walking, where adaptation and variability management are required. In addition, what defines the most suitable level of body weight support during gait training warrants further investigation, as body weight support inversely influences ground reaction forces, joint moments and subsequent muscle activations (43). Future studies should consider performing HAL-training over ground with well-balanced use of bodyweight support.

The lower overall positive joint work in the HAL group compared to the CONV group can be attributed to their somewhat slower walking speed (45). The vastly lower ankle work in both groups compared to controls may be attributed to reduced propulsive force during pre-swing, and corroborates reported findings of a significantly lower-than-normal positive ankle work in a stroke population at both self-selected and fast speeds (46). The greater proportion of hip and knee power in both groups compared to the normative dataset can be considered as a compensatory mechanism for the reduced ankle power (45, 47), and this indicates the importance of voluntary control in the hip and knee.

In most exoskeletons for walking, including HAL, the ankle joint does not articulate, restricting ankle motion and likely not encouraging voluntary ankle muscle activation. However, in normal gait, ankle motion is the largest contributor to forward propulsion (3). Recent studies on the use of a lightweight, soft, powered ankle exoskeleton (“exosuit”) in ambulatory chronic stroke patients have reported short term improvements during walking with the exosuit, i.e. more symmetrical and increased paretic forward propulsion, increased ankle dorsiflexion during swing phase, reduced energy cost (48), and reduced compensatory motions such as pelvic hiking and hip circumduction (49). This technology should be of interest in future studies including poorly- or non-ambulatory individuals in different stages after stroke, and evaluating the effect on gait patterns and walking after removing the exoskeleton.

In contrast to Baker et al. (28), who found a weak correlation between GPS and walking speed in a mixed population of patients and who suggested that both measures might be used to reflect different aspects of gait, we found these measures to have a moderate to good and significant correlation. This discrepancy is likely attributable to the slower walking speed in our study sample and suggests that kinematic gait deviations and walking speed might be more strongly correlated at slower walking speeds. It is worth noting that whereas patients in this study habitually used an ankle-foot orthosis and shoes when walking, they performed GA barefoot, which might have led to greater gait deviations than during their conventional gait training sessions.

The overall gait quality (GPS-LBP) was moderate-to-good and significantly correlated with independence in walking (FAC). The cognitive involvement associated with walking performance after stroke (50), partially incorporated in the FAC rating, and the use of compensatory strategies (42) might explain why the two are not more highly correlated. The GPS was previously found to increase when a cognitive task was implemented during over-ground walking (51) but to remain unchanged after robotic (end-effector) gait-training (52) in patients with Parkinson's disease.

We found a moderate correlation between overall gait quality (GPS-LBP) and movement function (FMA-LE Motor) in the present study. Previous studies in chronic stroke (53, 54), have suggested that the ability to perform isolated movements might not relate to complex motions during walking and might be insufficient to fully explain differences in walking performance and impaired gait pattern.

Impaired sensory function is known to contribute negatively to the probability of achieving independent gait (55) and to cause disturbed movements and asymmetric gait patterns (56). However, sensory function was not correlated to overall gait quality (GPS-LBP) in our study. Impaired sensory function might, however, negatively influence balance (57). Balance in standing (BBS) was correlated with overall gait quality in our study and has previously been suggested to be important for regaining walking ability after stroke (58). Future studies should consider assessing more advanced balance tasks such as non-level walking or multi-directional gait and their associations with gait quality.

It is study limitation that no patients were able to walk independently at inclusion and could not have adequately undergone a GA, thus making it impossible to compare changes in gait variables between intervention groups. It was also difficult to collect gait kinetics in the non-paretic limb in many patients, as walking aids frequently contacted the force plate. This consequently restricted between-leg comparisons of joint kinetics. It is worth noting that hemiparetic gait also has a large impact on kinematic and kinetics in the non-paretic side. Further, the non-disabled subjects were similar in age to the study sample but walked more quickly, which affected the GPS. Sample size calculation was based on the main study, as it was linked to prognoses of required study group sizes. Due to small sample size and high variability, the statistical power for this study is low, and the risk for type-2 errors is not negligible.

In the sub-acute phase after stroke, there appears to be a dose-response relationship (59) where increased practice of walking and activities related to walking results in better walking performance (i.e. walking ability, speed and activities of daily living) (60). However, as presented above, the HAL group performed more gait training sessions in total, but fewer conventional gait training sessions. During the conventional gait training the distance walked by the HAL group was also shorter compared to the CONV group. The overall dose, intensity and variability of gait training may have affected our results and should be considered in future studies (13).

Lastly, participants in the current study were younger and more severely disabled that the overall stroke population, which should be taken into consideration when generalizing our findings. However, this group of patients warrants attention for these same reasons and thus might also face long-term disability. This study does not provide any firm conclusions, but it provides important information and a rationale for larger studies.

## Conclusions

We found that the addition of HAL-training to evidence-based conventional training did not improve the outcome in terms of gait deviation, spatiotemporal asymmetry, or positive joint work in this small, relatively young and severely impaired stroke population. The overall gait quality was correlated with independence in walking, walking speed, balance and movement function, but not with sensory function. Though of limited scope, this study suggests that retraining of a normal gait pattern might not be attainable by conventional gait training according to today's standards nor by electromechanically-assisted gait training in patients with severe limitations in walking. This raises new questions regarding how post-stroke rehabilitation programs, with or without electromechanically-assisted gait training, for patients with moderate to severe gait impairments should be designed in order to further improve both walking ability and recovery of gait pattern functions. Findings from this study may be useful in planning of future larger studies exploring whether electromechanically-assisted gait training can improve gait function, and if so, in whom.

## Data availability statement

The raw data supporting the conclusions of this article will be made available by the authors, without undue reservation.

## Ethics statement

The studies involving humans were approved by Swedish Ethical Review Authority. The studies were conducted in accordance with the local legislation and institutional requirements. The participants provided their written informed consent to participate in this study. Written informed consent was obtained from the individual(s) for the publication of any potentially identifiable images or data included in this article.

## Author contributions

AW, SP, JB, and EG-F participated in the study design and drafted and provided important intellectual content to the manuscript. AW coordinated the data collection. AW and EG-F performed gait analyses and including data processing. AW performed the statistical analysis in cooperation with SP. All authors have approved the final manuscript.
