# Supplementary material for: Gait pattern after electromechanically-assisted gait training with the Hybrid Assistive Limb and conventional gait training in sub-acute stroke rehabilitation—A subsample from a randomized controlled trial
Source: Front Neurol. 2023 Oct 11;14:1244287. doi: 10.3389/fneur.2023.1244287 (PMC10598624; doi:10.3389/fneur.2023.1244287)
Supplement: Supplementary file 1 [file Data_Sheet_1.PDF]

SUPPLEMENTARY MATERIAL

Three-dimensional kinematics, specifically pelvis, hip, knee, ankle and foot angles, are illustrated from one gait cycle in each patient in Supplementary Figure 1 for (a) CONV and (b) HAL intervention groups. Kinetics, specifically hip, knee and ankle moments in three dimensions, as well as joint power, from one gait cycle in each patient are illustrated in Supplementary Figure 2 for (a) CONV and (b) HAL intervention groups.

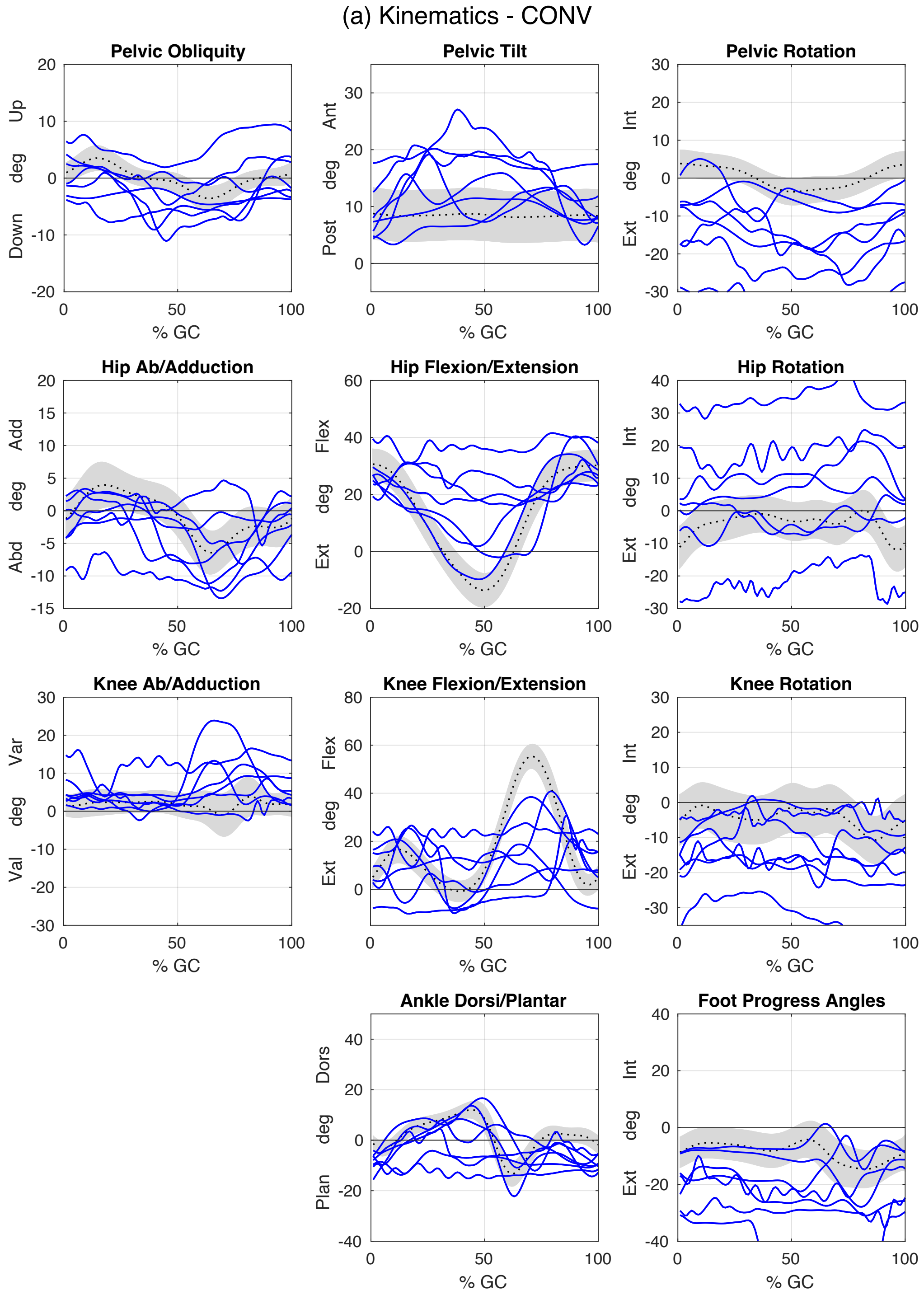

(a) Kinematics - CONV
Pelvic Obliquity
Up
deg
Down
% GC
Pelvic Tilt
Ant
deg
Post
% GC
Pelvic Rotation
Int
deg
Ext
% GC
Hip Ab/Adduction
Add
deg
Abd
% GC
Hip Flexion/Extension
Flex
deg
Ext
% GC
Hip Rotation
Int
deg
Ext
% GC
Knee Ab/Adduction
Var
deg
Val
% GC
Knee Flexion/Extension
Flex
deg
Ext
% GC
Knee Rotation
Int
deg
Ext
% GC
Ankle Dorsi/Plantar
Dors
deg
Plan
% GC
Foot Progress Angles
Int
deg
Ext
% GC

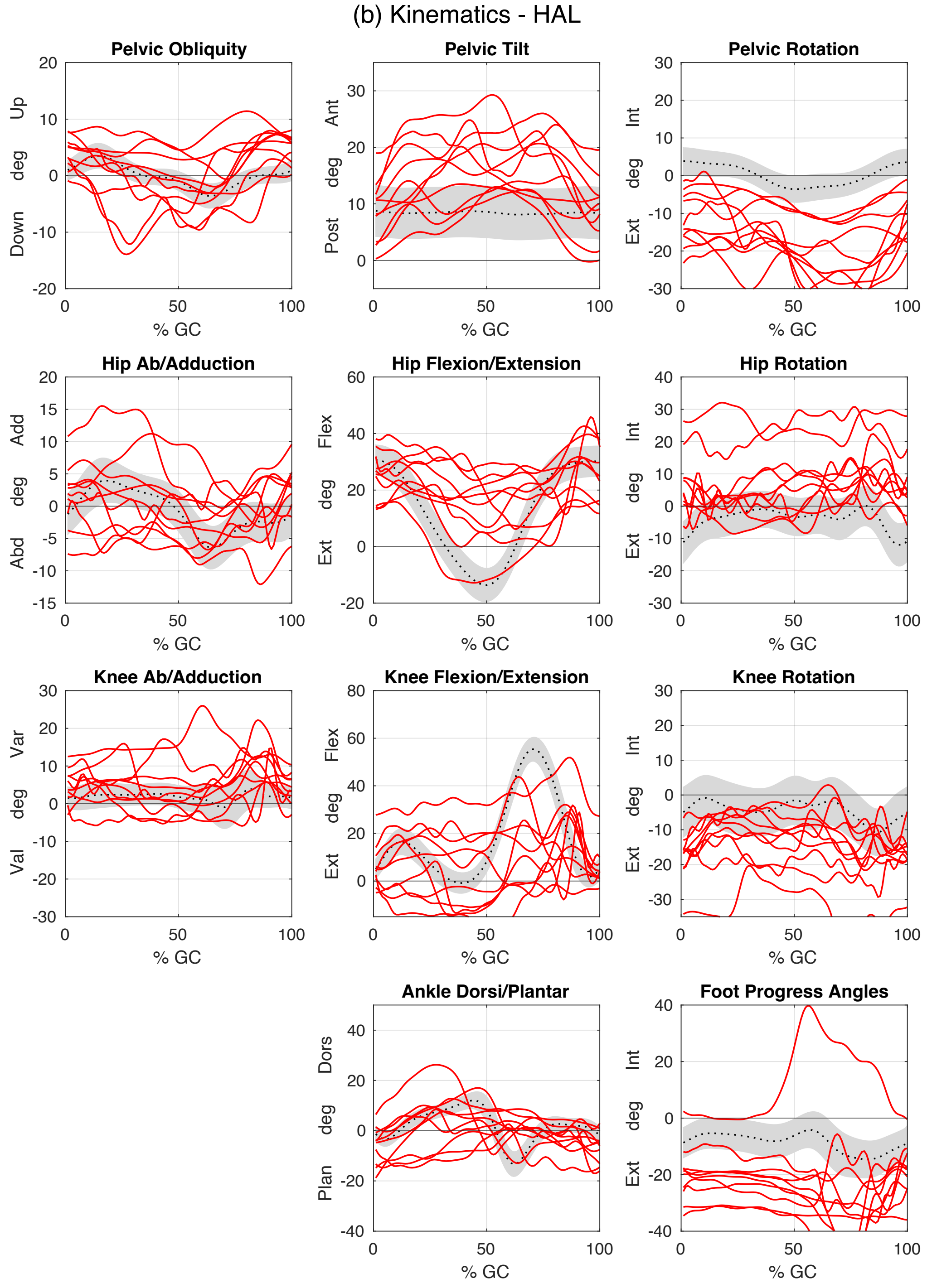

Supplementary Figure 1: Kinematics during one gait cycle in the paretic side for each patient in the (a) CONV (*n*=7) group and (b) HAL (*n*=10) group. The normative dataset ± 1 standard deviation is shown in gray.

(a) Moments and Power - CONV

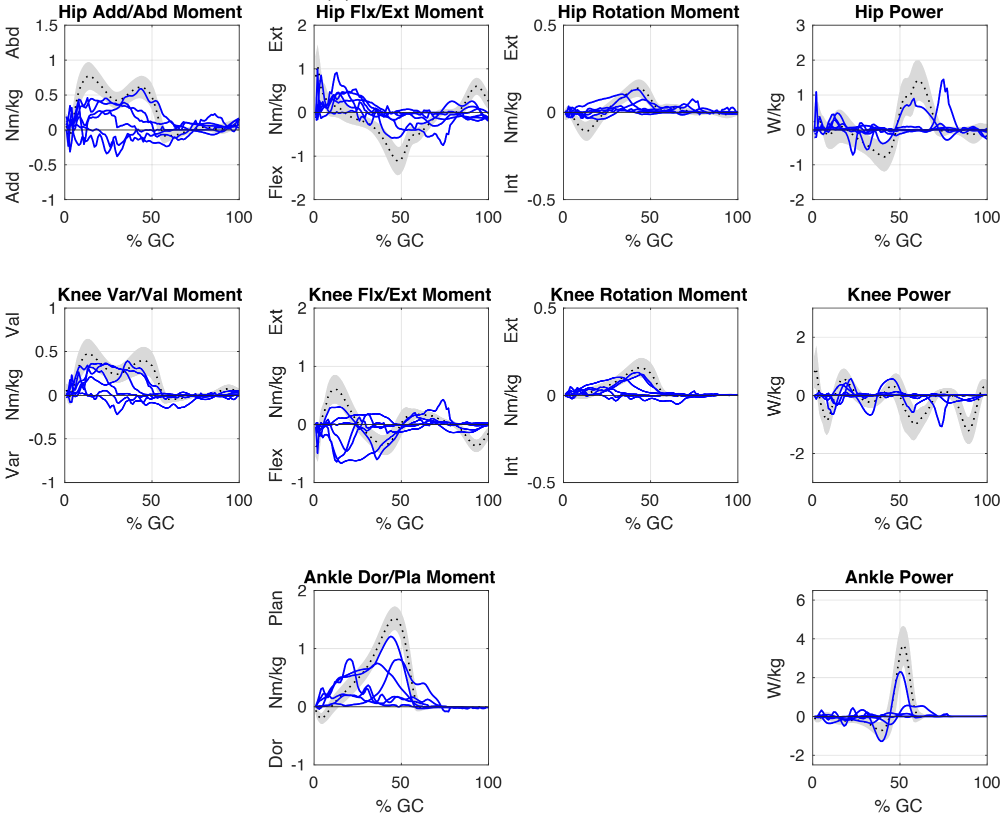

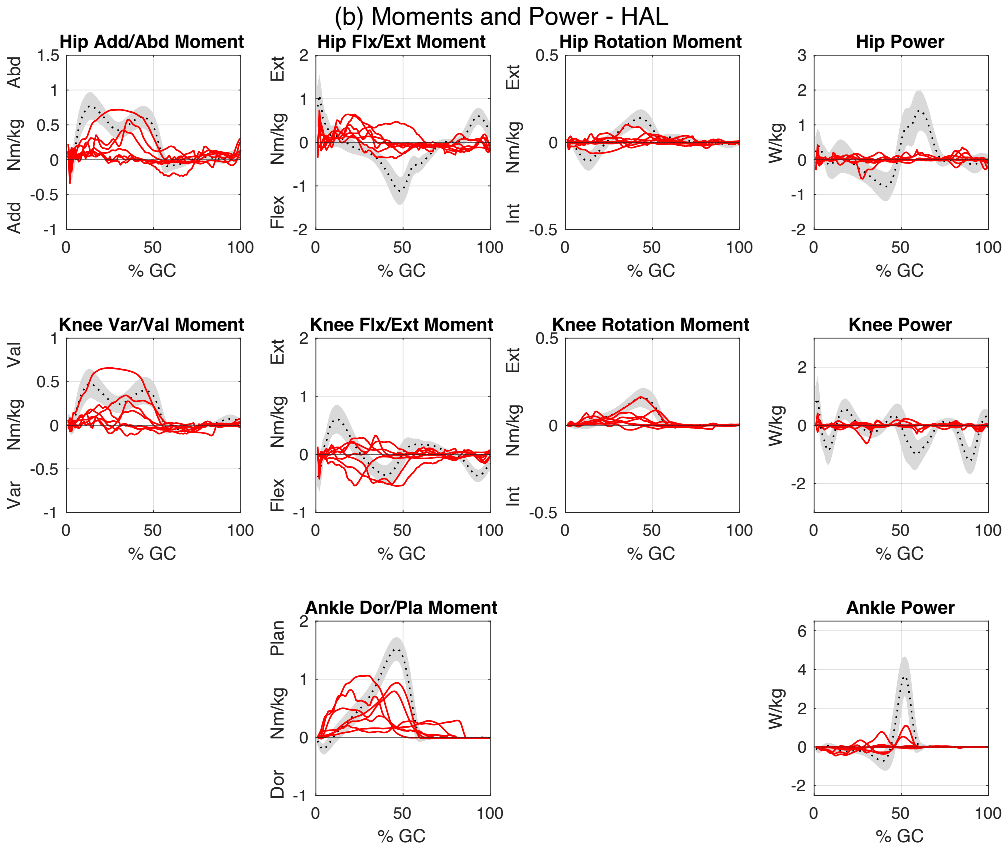

Supplementary Figure 2: Kinetics during one gait cycle in the paretic side for each patient in the (a) CONV (*n*=6) group and (b) HAL (*n*=7) group for which gait kinetics were collected. The normative dataset ± 1 standard deviation is shown in gray.
